# Human papillomavirus and oropharyngeal cancer in Greenland in 1994–2010

**DOI:** 10.3402/ijch.v72i0.22386

**Published:** 2013-11-06

**Authors:** Magnus Balslev Avnstorp, Ramon Gordon Jensen, Emilie Garnæs, Marianne Hamilton Therkildsen, Bodil Norrild, Lena Specht, Christian von Buchwald, Preben Homøe

**Affiliations:** 1 Department of Otolaryngology, Head & Neck Surgery, Rigshospitalet, University of Copenhagen, Denmark; 2 Department of Pathology, Rigshospitalet, University of Copenhagen, Denmark; 3 Department of Cellular & Molecular Medicine, Panum Instituttet, University of Copenhagen, Denmark; 4 Department of Oncology, Rigshospitalet, University of Copenhagen, Denmark

**Keywords:** head and neck cancer, HPV, Inuit, p16 immunohistochemistry, PCR, HPV vaccine, immunization

## Abstract

**Background:**

Oropharyngeal squamous cell carcinoma (OPSCC) is associated with the sexually transmitted human papillomavirus (HPV), smoking and alcohol. In Greenland, a high rate of HPV-induced cervical cancer and venereal diseases are found, which exposes the population for high risk of HPV infection. In Greenland, only girls are included in the mandatory HPV vaccination program.

**Objective:**

To investigate the annual incidence of OPSCC and the proportion of HPV-associated OPSCC (HPV+ OPSCC) in Greenland in 1994–2010.

**Design:**

At Rigshospitalet, University of Copenhagen, we identified all Greenlandic patients diagnosed and treated for OPSCC from 1994 to 2010. Sections were cut from the patient's paraffin-embedded tissue blocks and investigated for p16 expression by immunohistochemistry. HPV analyses were performed with 2 sets of general HPV primers and 1 set of HPV16-specific primer. HPV+ OPSCC was defined as both >75% p16+ cells and PCR positive for HPV.

**Results:**

Of 26 Greenlandic patients diagnosed with OPSCC, 17 were males and 9 were females. The proportion of HPV+ OPSCC in the total study period was 22%, without significant changes in the population in Greenland. We found an increase in the proportion of HPV+ OPSCC from 14% in 1994–2001 to 25% in 2002–2010 (p=0.51). Among males from 20 to 27% (p=0.63) and in females from 0 to 20% (p=0.71). The annual OPSCC incidence increased from 2.3/100,000 (CI=1.2–4.2) in 1994–2001 to 3.8/100,000 (CI=2.4–6.2) in 2002–2010: among males from 2.4/100,000 (CI=1.0–5.7) to 5.0/100,000 (CI=2.9–8.9).

**Conclusion:**

Even though the population is at high risk of HPV infection, the proportion of 22% HPV+ OPSCC in the total study period is low compared to Europe and the United States. This might be explained by our small study size and/or by ethnic, geographical, sexual and cultural differences. Continuing observations of the OPSCC incidence and the proportion of HPV+ OPSCC in Greenland are needed.

Head and neck cancer is the fourth most common malignant cancer worldwide and is known to be associated with high consumption of alcohol, tobacco and human papillomavirus infection (HPV) (1, 2). Incidence of oropharyngeal squamous cell carcinoma (OPSCC), located in the tonsils, the tongue base and the soft palate has increased, and it is now the most frequent head and neck cancer in the United States (3). In Denmark, the incidence of OPSCC has increased from a rate of 1.5/100,000 in 1970 to a rate of 5/100,000 in 2010 (4). The increase tended to be in younger males and is hypothesized to be caused by sexually transmitted HPV (5).

In Greenland, the annual incidence rate of OPSCC from 1994 to 2003 was 2.1/100,000 accounting for 11% of all head and neck cancers in Greenland (6). The development of OPSCC in Greenland is thought to be explained by high tobacco consumption (79% smokers in 1993–1994), alcohol consumption and possibly by HPV infection (6, 7). However, to date no investigation of the proportion of HPV-associated OPSCC (HPV+ OPSCC) in Greenland has been conducted (6, 8). In Greenland, the patients have often been diagnosed late in advanced stages (69% in stage III–IV), with a low 5-year survival rate from OPSCC in 1994–2003 of 30%, compared to 51% for tongue base cancer, and 60% for tonsillar cancer in the United States in 2000–2002 (6, 9). A lower survival rate in Greenland may be remedied by HPV immunization and improved treatment of OPSCC. Determining the proportion of vaccine-preventable HPV+ OPSCC in Greenland is needed.

HPV is known to cause cervical squamous cell carcinoma (CSCC). A recent study finds a significant similarity in the miRNA profile of HPV+ CSCC and HPV+ OPSCC (10). Other recent studies suggest an association of HPV with squamous cell carcinomas such as anal, penile and even breast cancer, which have similar non-keratinizing epithelium as the cervix (11, 12). In Greenland, the annual incidence rate of CSCC from 1988 to 1996 was three to four times higher than the rate in Denmark, the age of sexual debut was lower, and a higher incidence of venereal diseases such as gonorrhoea was found (13, 14). These facts and a register study reporting that partners to women with CSCC have an increased risk of acquiring HPV+ OPSCC, indicate a population with a high risk of sexually transmitted HPV, that could possibly lead to HPV+ OPSCC (15).

The carcinogenic effect of HPV is caused by the expressed oncoproteins E6 and E7 that inactivate the tumour suppressor genes p53 and retinoblastoma protein (pRB) leading to uncontrolled growth and a reciprocal up-regulation of the tumour suppressor protein p16 (16, 17). p16 is therefore considered as a surrogate marker for HPV infection. The specific HPV type can be determined using a DNA-based PCR analysis on tumour DNA (18). More than 100 types of HPV are known, but only the 15 types HPV16, 18, 31, 33, 35, 39, 45, 51, 52, 56, 58, 59, 68, 73 and 82 are of high risk and carcinogenic (19). In Greenland, 96.3% of the HPV+ CSCC was found to be HPV16 induced, and we therefore expect most of the HPV+ OPSCC to be induced by HPV16 (20).

In Greenland, the HPV vaccine is currently administered, free of charge, only to girls aged 12–27 years as a part of the Danish and Greenlandic free mandatory national vaccination programme (21). Introduction of the HPV vaccine to males in Greenland has been discussed, but the government has chosen not do so yet.

The aims of the present study are to investigate the proportion of HPV+ OPSCC and OPSCC incidence in the Greenlandic population and to find potential changes in the time period 1994–2001 compared to 2002–2010. The perspectives of HPV vaccination of boys will also be presented.

## Patients and methods

### Patients

This study is retrospective, including patients born and living in Greenland who were biopsied and diagnosed with OPSCC (ICD-code C05.1, C09, C10 and C14.2) in the period 1994–2010. The patients were biopsied in Greenland or at Rigshospitalet, and all were transferred for further treatment at Rigshospitalet, which serves as a tertiary referral hospital for Greenland. The patients and their medical records were identified by the patient registry at Rigshospitalet and the diagnosis verified by the Danish Pathology Registry. Information regarding tobacco and alcohol consumption, and the stage of cancer was obtained from medical records and from the Danish Head and Neck Cancer Group database (DAHANCA) (22). The patient's birthplace in Greenland was validated by a workgroup on Statens Serum Institut (SSI), by finding the patient in the national birthplace database in Greenland using the patient's unique civil registration system number.

### p16 immunohistochemistry procedure

**Fig. 1:**
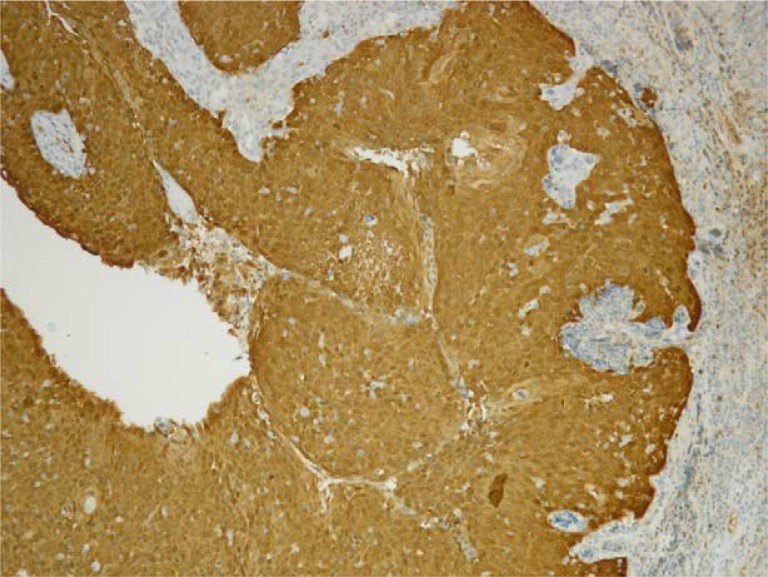
p16+ immunohistochemistry staining (>75% positive brown coloured cells) on oropharyngeal squamous cell carcinoma (OPSCC) section.

Formalin-fixed paraffin-embedded (FFPE) tumour tissue blocks (archival specimens) of OPSCC from the Greenlandic patients were collected at the Department of Pathology, Rigshospitalet, where all histological specimens from Greenland are examined. Tumour sections of 4 µm were cut, stained with HE and the OPSCC diagnosis was verified by a specialized head and neck pathologist. For p16 immunohistochemistry, the representative slides were incubated with p16 antibody (JC8, Santa Cruz) and stained on a BenchMark ULTRA (Roche, Copenhagen, Denmark) using the UltraView detection system (Fig. 1). The p16 staining results were scored by the same specialized pathologist as: 0=no positive staining, 1+=1–25%, 2+=26–50%, 3+=51–75% and 4+=76–100%. Only the slides with the score 4+ (>75% staining) and staining of both the nuclei and cytoplasm were considered p16 positive (p16+).

### PCR analysis

Four sections of 10 µm were cut from the FFPE OPSCC specimens and were transferred to Eppendorf tubes. The genomic DNA was extracted by the QIAamp procedure (23) after pretreatment of the sections with xylene and precipitation with 100% EtOH. Two DNA extractions were performed on sections from each patient and analyzed for purity, mass and quantification of DNA by Nanodrop equipment (24). Afterwards, the extracted samples were stored at −20°C until the PCR procedure was performed.

**Fig. 2:**
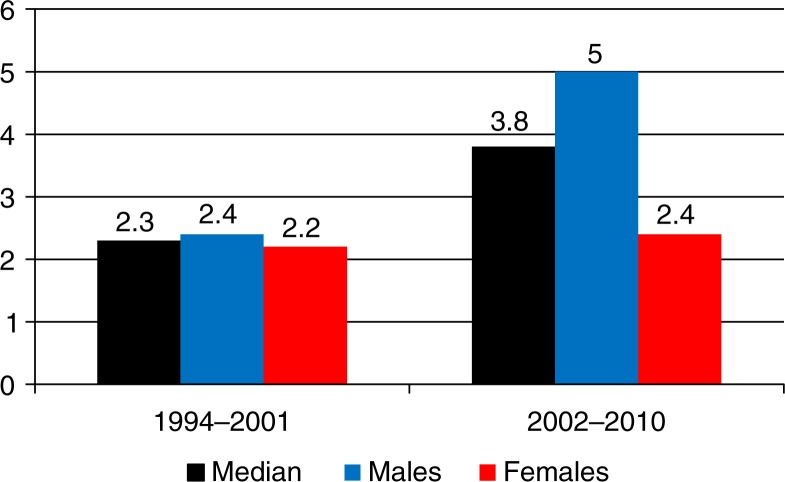
Oropharyngeal squamous cell carcinoma (OPSCC) incidence per 100,000 Greenlandic inhabitants in 1994–2001 and 2002–2010.

**Fig. 3:**
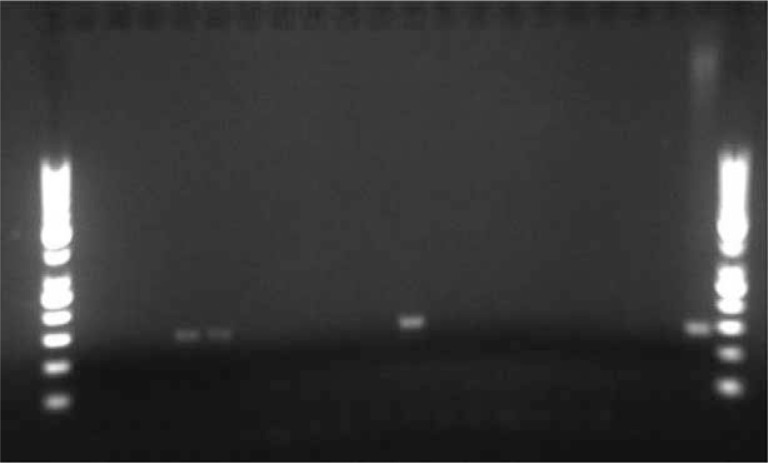
Three PCR-positive oropharyngeal squamous cell carcinoma (OPSCC) cases and 1 PCR-positive control on agarose gel.

DNA quality of the extracted DNA samples was controlled by the expression of the housekeeping gene glyceraldehyde 3-phosptate dehydrogenase (GAPDH) (25). Only DNA samples expressing GAPDH were used for HPV-specific PCR analysis. Two general primer sets My09/My11, Gp5+/Gp6+ and 1 set of specific HPV16 primers were used as described by Lajer et al. (10). If one of the primer sets amplified the HPV DNA and was visualized on 2.5% agarose gel, the sample was scored as PCR positive for HPV (Fig. 3).

### Definition of HPV-associated (HPV+) OPSCC

We defined HPV-associated (HPV+) OPSCC by 2 criteria: The p16 immunohistochemistry should be of score 4+ (>75% positive cells) and the specimen should be PCR positive with one of the HPV-specific primer sets for OPSCC (26). As smoking down-regulates p16 expression, this rigorous definition was applied to clarify the OPSCC that was induced by HPV (27).

### Statistics

Statistical analyses were performed using SPSS v. 20 from IBM. Fisher's exact test was used for testing proportions. Significant results were defined as a p-value of ≤0.05. Annual incidence rates were age-standardized using the world population and calculated using the mean population of Greenlandic inhabitants as the denominator in 2 study time intervals: 1994–2001 (48,924 people, males 26,193, females 22,731) and 2002–2010 (49,618 people, males 26,593, females 23,025) (28). Rothman/Greenland method was used for calculating 95% confidence intervals in Openepi (29).

## Ethics

The study followed the Helsinki II declaration and was approved by the Commission for Scientific Research in Greenland. The Danish Data Protection Agency approved the use of the patient's data.

## Results

A total of 30 patients diagnosed with OPSCC in Greenland from 1994 to 2010 were identified. Of these, 4 patients were not born in Greenland, leaving 26 ethnical Greenlanders with available OPSCC specimens for the study. Table I shows the annual incidence rate of OPSCC in the study period, the proportion of p16+ OPSCC, HPV+ OPSCC and HPV− OPSCC according to sex distribution, the time intervals 1994–2001 and 2002–2010, smoking habits and alcohol intake. Table II shows the median age when diagnosed with OPSCC according to p16+ OPSCC, HPV+ OPSCC, HPV− OPSCC and the gender of the patients.

**Table I:** Characteristics for Greenlandic OPSCC patients in 1994–2010

	n (IR per 100,000 or %)	p16+ (%)	HPV+ (%)§	HPV− (%)§
Total study period 1994–2010	26 (3.1)	11/26 (42)	5/23 (22)	18/23 (78)
Male	17 (3.8)	8/17 (47)	4/16 (25)	12/16 (75)
Female	9 (2.3)	3/9 (33)	1/7 (14)	6/7 (86)
1994–2001	9 (2.3)	2/9 (22)	1/7 (14)	6/7 (86)
Male	5 (2.4)	2/5 (40)	1/5 (20)	4/5 (80)
Female	4 (2.2)	0/4 (0)	0/2 (0)	2/2 (100)
2002–2010	17 (3.8)	9/17 (53)	4/16 (25)	12/16 (75)
Male	12 (5.0)	7/12 (58)	3/11 (27)	7/11 (64)
Female	5 (2.4)	2/5 (40)	1/5 (20)	4/5 (80)
Smokers (current or former)†	17/21 (81)	7/9 (78)	2/4 (50)	15/17 (88)
Male	12/14 (86)	6/7 (86)	2/3 (66)	10/11 (91)
Female	5/7 (71)	1/2 (50)	0/1 (0)	5/6 (83)
Alcohol consumption (>7 units/week)†	13/21 (62)	5/8 (63)	1/4 (25)	12/17 (71)
Male	10/14 (71)	4/6 (71)	1/3 (33)	9/11 (82)
Female	3/7 (43)	1/2 (50)	0/1 (0)	3/6 (50)

p16+:>75% p16-positive cells by immunohistochemistry; HPV+: p16+ *and* PCR-positive; HPV−: negative for p16 *and/or* PCR;

§ Of 23 GAPDH-positve OPSCC cases

† Data on smoking and alcohol available for 21 patients.

OPSCC, oropharyngeal squamous cell carcinoma.

**Table II:** Median age and characteristics for Greenlandic OPSCC patients in 1994–2010

	Total [range]	p16+ [range]	HPV+ [range]	HPV− [range]
Total	63 [39–81]	62 [39–81]	47 [39–73]	63 [50–81]
Male	63 [39–77]	61 [39–77]	57 [39–73]	62 [52–77]
Female	66 [47–81]	68 [47–81]	47 [–]	68 [50–81]

p16+:>75% positive cells by p16 immunohistochemistry. HPV+: both PCR-positive *and* p16+; HPV−: negative for PCR *and/or* p16.

### p16 immunohistochemistry and PCR analysis

In the total study period 1994–2010, we found 11/26 (42%) of the OPSCC specimens to be p16+ (score 4+, >75% staining), while 3/26 (12%) were of score 1+ and 2+ (<50% staining). The rest of the OPSCC specimens presented no p16 staining. Table I shows results of p16+ OPSCC according to the sex distribution and time intervals. PCR analysis showed that 88% (n=23) of the OPSCC specimens were positive for the housekeeping gene GAPDH and therefore were suitable for HPV-specific PCR analysis. Of these 5/23 (22%) were PCR positive. All PCR-positive OPSCC specimens were p16+ of score 4+, which resulted in an overall proportion of 22% HPV+ OPSCCs. In males, 4/16 (25%) of OPSCC were HPV+, compared to 1/7 (14%) in females (p=0.5) (Table I). Four out of 5 HPV+ OPSCC were of the type HPV16.

The median age at diagnosis of the patients with HPV+ OPSCC was 47 years compared to the age of 63 years when diagnosed with HPV− OPSCC (p=0.3) (Table II).

### Study time interval 1994–2001 compared to 2002–2010

In 1994–2001, the annual OPSCC incidence was 2.3/100,000 (CI=1.2–4.2), in males 2.4/100,000 (CI=1.0–5.7) and in females 2.2/100,000 (CI=0.8–5.8) (Table I). The annual OPSCC incidence in 2002–2010 increased to 3.8/100,000 (CI=2.4–6.2), in males to 5.0/100,000 (CI=2.9–8.9) and in females to 2.4/100,000 (CI=1.0–5.8) (Fig. 2 and Table I).

In 1994–2001, the proportion of p16+ OPSCC was 22% (40% in males and 0% in females) and the proportion of HPV+ OPSCC was 14% (20% in males and 0% in females). In 2002–2010, the proportion of p16+ increased to 53% (p=0.14), in males to 58% (p=0.56) and in females to 40% (p=0.12), while the proportion of HPV+ OPSCC increased to 25% (p=0.51), in males to 27% (p=0.63), and in females to 20% (p=0.71).

### Tobacco and alcohol

Data on tobacco and alcohol consumption were available for 21 of the 26 OPSCC patients. Of the HPV+ OPSCC patients, 2/4 (50%) were smokers and 1/4 (25%) consumed more than 7 units of alcohol per week, while among the HPV− OPSCC patients, 15/17 (88%) were smokers (p=0.15) and 12/17 (71%) consumed more than 7 units per week (p=0.13) (Table I).

## Discussion

In our study, we found an increase in the proportion of HPV+ OPSCC from 14% in 1994–2001 to 25% in 2002–2010, and an overall HPV+ OPSCC proportion of 22%. Compared to Europe, the proportion is low. A study conducted in the United Kingdom using similar methods and definitions of HPV+ examined 108 OPSCC patients and found an increase in the proportion of HPV+ OPSCC from 15% in 1988 to 57% in 2009 (30). International meta-analyses and reviews have found the proportion of HPV+ OPSCC to be between 20 and 75%, increasing the last decades (5, 12, 31). The genetic and cultural aspects may play a role, since a study conducted in Hokkaido, Japan, using solely the sensible PCR method, including 71 OPSCC patients from 1998 to 2009 found the proportion of HPV+ OPSCC to be only 32% (32).

Our lower proportion of 22% HPV+ OPSCC, compared to western countries, may be explained by the small study group consisting of only 26 Greenlandic patients. Also high smoking and alcohol consumption was so dominant in Greenland that the effect of the HPV maybe is difficult to observe (Table I). In 1993–1994, the prevalence of current smokers was 79% in Greenland and decreased to 66% in 2005. As this prevalence is expected to further decrease, the proportion of HPV+ OPSCC must be expected to increase.

It is known that HPV is sexually transmitted and the rate of oral infection with HPV16 correlates with the number of sexual partners, oral sex and open-mouthed kissing. Also, from a register study we know that partners to women with positive pap-smears for CSCC are at higher risk of OPSCC (15, 33). The Greenlandic inhabitants have an early sexual debut, high rates of venereal disease and 3–4 times the CSCC rate of Denmark, but surprisingly this does not seem to have induced a high proportion of HPV+ OPSCC in Greenland at present (13, 14, 20). The findings correlates with a study from 2008 that found the prevalence of cervical HPV infection to be lower in Greenlandic women aged more than 20 years, compared to Danish women (34). The lower proportion of HPV+ OPSCC could be explained by different sexual practice, hygiene, genetics, cultural differences or maybe because the spread of HPV started 10 years later in Greenland (34). These theories need further investigation.

The proportion of 22% HPV+ OPSCC cases demonstrates that HPV infection is more common in the Greenlandic patients with OPSCC compared to healthy people. American studies conducted in 2009–2010 among 3,977 healthy Americans found 1.3% to have oral HPV infection (35), and another study found that 3.6% of females and 10% of males had some type of oral HPV infection (36). The proportion of HPV infection in the American study was found to correlate positively with the number of sexual partners (36). The proportion of oral HPV infection in healthy individuals still needs to be investigated in Greenland.

We find a discrepancy between the proportion of 42% p16+ OPSCC cases and only 22% PCR-positive OPSCC cases. These p16+ and PCR-negative OPSCC cases may have been induced by other factors such as other viral infections or mutations (e.g. adenovirus, CMV or Polyoma SV40 virus) that might inactivate pRB and thereby lead to an up-regulation of p16 (37, 38). p16 up-regulation may also be induced be cellular oxidative stress, cell ageing or physiological stress (39–41). To exclude laboratory bias and older eventually degraded specimens, we controlled the DNA quality by the Nanodrop procedure and by PCR for the housekeeping gene GAPDH before performing PCR for HPV (24, 25, 42).

Males accounted for two-thirds of the OPSCC cases. The alcohol and tobacco consumption were lower in patients with HPV+ OPSCC compared to HPV− OPSCC patients, and the HPV+ OPSCC cases were diagnosed 16 years earlier (median 47 years) compared to HPV− OPSCC (median 63 years). These results are in accordance with international findings (5, 43, 44).

## Perspectives

Possible treatments of HPV+ OPSCC today include surgery and radiotherapy with or without concomitant chemotherapy. But it is possible that vaccines administered today will prevent HPV+ OPSCC in the future. The quadrivalent vaccine covers the oncogenic HPV16 and HPV18 viruses and the non-oncogenic, papilloma inducing HPV6 and HPV11 viruses, and the bivalent vaccine covers HPV16 and HPV18 viruses. In a multi-ethnic, multinational (12, 45) 4-year follow-up study and in a Danish study (46), the quadrivalent vaccine was shown to be effective against HPV6, 11, 16 and 18, with fewer anal papillomas and cervical squamous cell neoplasias and with continuously high anti-HPV titres in the blood.

From 1988 to 2004, the annual incidence of HPV+ OPSCC in the United States has increased 225%, and it is especially found in males aged 30–50. The incidence of HPV+ OPSCC is predicted to surpass the incidence of HPV+ CSCC in the year 2020 (5, 47). As a response to this and the fact that HPV is associated with anal and penile cancer, the US Food and Drug Administration in 2009–2011 approved the HPV vaccine to both girls and boys aged 9–16 years (11, 12, 45). In Greenland, the vaccine is also approved for boys, but not included in the mandatory childhood vaccination programme (21). Currently, the proportion of HPV+ OPSCC is low in Greenland, but an increase in HPV+ OPSCC may occur in the future. Continuing studies of the proportion and incidence of HPV+ OPSCC are important for the consideration of including HPV vaccination for boys in the mandatory childhood vaccination programme.

## Conclusion

We found an increase in the annual OPSCC incidence in Greenland from 2.3/100,000 (CI=1.2–4.2) in 1994–2000 to 3.8/100,000 (CI=2.4–6.2) in 2001–2010; among males from 2.4/100,000 (CI=1.0–5.7) to 5.0/100,000 (CI=2.9–8.9). We found an increase in the proportion of HPV+ OPSCC from 14 to 25% in the same interval (p=0.51). Patients suffering from HPV+ OPSCC were diagnosed at an earlier age (47 years compared to 63 years), and there was a trend towards lower consumption of alcohol (p=0.13) and tobacco (p=0.15) compared to patients with HPV− OPSCC. The overall proportion of 22% HPV+ OPSCC was lower compared to Europe and the United States, possibly due to small sample sizes and/or geographical, sexual, ethnic or cultural differences. The Greenlandic population is still at high risk of HPV infection as demonstrated by the high incidence of HPV-induced CSCC. Continuing studies of the OPSCC incidence and the proportion of HPV+ OPSCC in Greenland are needed.
